# Acute anxiety and social inference: An experimental manipulation with 7.5% carbon dioxide inhalation

**DOI:** 10.1177/0269881116653105

**Published:** 2016-07-04

**Authors:** Katherine S Button, Lucy Karwatowska, Daphne Kounali, Marcus R Munafò, Angela S Attwood

**Affiliations:** 1 Department of Psychology, University of Bath, Calverton Down, Bath, UK; 2 UK Centre for Tobacco and Alcohol Studies, School of Experimental Psychology, University of Bristol, Bristol, UK; 3 School of Social and Community Medicine, University of Bristol, Bristol, UK; 4 Integrative Epidemiology Unit, Medical Research Council, University of Bristol, Bristol, UK

**Keywords:** Anxiety, carbon dioxide inhalation, carbon dioxide levels, learning, negativity, positivity, self-perception, self-referential processing, social anxiety, social judgement, state anxiety, trait anxiety

## Abstract

**Background:**

Positive self-bias is thought to be protective for mental health. We previously found that the degree of positive bias when learning self-referential social evaluation decreases with increasing social anxiety. It is unclear whether this reduction is driven by differences in state or trait anxiety, as both are elevated in social anxiety; therefore, we examined the effects on the state of anxiety induced by the 7.5% carbon dioxide (CO_2_) inhalation model of generalised anxiety disorder (GAD) on social evaluation learning.

**Methods:**

For our study, 48 (24 of female gender) healthy volunteers took two inhalations (medical air and 7.5% CO_2_, counterbalanced) whilst learning social rules (self-like, self-dislike, other-like and other-dislike) in an instrumental social evaluation learning task. We analysed the outcomes (number of positive responses and errors to criterion) using the random effects Poisson regression.

**Results:**

Participants made fewer and more positive responses when breathing 7.5% CO_2_ in the other-like and other-dislike rules, respectively (gas × condition × rule interaction *p* = 0.03). Individuals made fewer errors learning self-like than self-dislike, and this positive self-bias was unaffected by CO_2_. Breathing 7.5% CO_2_ increased errors, but only in the other-referential rules (gas × condition × rule interaction *p* = 0.003).

**Conclusions:**

Positive self-bias (i.e. fewer errors learning self-like than self-dislike) seemed robust to changes in state anxiety. In contrast, learning other-referential evaluation was impaired as state anxiety increased. This suggested that the previously observed variations in self-bias arise due to trait, rather than state, characteristics.

## Introduction

Humans rapidly learn the social salience of stimuli (Sui et al., 2013), and the cognitive mechanisms supporting this learning are often strongly self-biased (Humphreys and Sui, 2015). Furthermore, individuals tend to show a robust preference for positive, as opposed to negative, self-information (Sharot, 2011); and this self-optimism is thought to be protective for mental health (Korn et al., 2012; Taylor and Brown, 1988, 1994b). Importantly, reduced positive self-bias is implicated in depression (often referred to as ‘depressive realism’, as in a recent review (Moore and Fresco, 2012)) and more recently, in social anxiety (Button et al., 2012, 2015).

During a social interaction, individuals use accruing social cues to infer what the other person is thinking (e.g. ‘Do they like me?’ or ‘Do they agree with what I’ve just said?’). Social interactions are therefore dynamic, with social behaviours contingent on evaluative feedback, which is often expressed via ambiguous social cues. Thus, reinforcement or instrumental learning may be a particularly important mechanism in maintaining positive self-bias during and after social interaction; and disruptions to, or biases in, this mechanism may contribute to social anxiety and depression (Button et al., 2012, 2015; Korn et al., 2012, 2014). For example, individuals with low anxiety make fewer errors learning positive rather than negative self-referential evaluation, and, based on the same level of objective evidence, rate themselves as being more positively evaluated than others ( Button et al., 2012, 2015; De Jong, 2002). Selectively disregarding evidence of negative self-referential evaluation would presumably increase confidence during social interactions, produce better social outcomes and reduce the availability of negative information for later rumination. This may be one way a positive self-bias protects mental health.

Social anxiety is characterised by trait fears of being negatively evaluated by others (Diagnostic and Statistical Manual of Mental Disorders, version 5 (DSM-5)). Cognitive models of social anxiety emphasise the role of negative self-schema, sets of deeply held beliefs about the self (e.g. ‘I am unlikable’), the world (e.g. ‘Social interactions are threatening as others are highly critical’) and the future (e.g. ‘It will always be this bad’), in maintaining these disorders (Clark and Wells, 1995; Rapee and Heimberg, 1997). Once activated, these schema are thought to bias processing of stimuli in a negative way, giving rise to negative interpretations of social experiences, which in turn lead to rumination and social avoidance (Abbott and Rapee, 2004).

Dual process models functionally distinguish between the automatic processes occurring during a social interaction and the more reflective processing involved in anticipation and post-event rumination, which might occur before or afterwards (Strack and Deutsch, 2004). In support of cognitive models (Clark and Wells, 1995), there is good evidence of negative biases in reflective processes in social anxiety. For example, socially anxious individuals are more inclined to negative interpretations of ambiguous social vignettes (Amin et al., 1998; Hirsch and Mathews, 1997). however, evidence from paradigms tapping more automatic processes, such as associative or instrumental learning, suggests that rather than being negatively biased, socially anxious individuals show a reduction in positive self-bias relative to controls (Button et al., 2012, 2015; De Jong et al., 2009; Garner et al., 2006; Hirsh and Mathews, 2000).

To examine the role of self-biased social-evaluative learning in social anxiety, and how this relates to post-event reflective processing, we developed an instrumental learning task (Button et al., 2015). This task required participants to learn social-evaluative rules (‘I am liked’, ‘I am disliked’, ‘Other is liked’ or ‘Other is disliked’), by selecting words in a series of positive or negative word pairs that fitted most with what a computer persona thought about themself or a fictional third person, George (the ‘other’).

The persona provided feedback (i.e. correct or incorrect); and the participants used trial and error over a series of trials, to infer how they or George were evaluated. At the end each rule, the participants were asked to provide a global rating. Therefore, the task assessed both learning rates and global interpretations for each rule (Button et al., 2015).

The least anxious showed a strong bias for positive words. We found evidence of a reduction in this positive bias in social anxiety, that was:

1. Specific to cues relating to the self and not others (evidence for reduced positive self-bias);
2. Strongest for negative self-evaluation (consistent with core fears of negative evaluation in social anxiety); and
3. Predicted negatively-biased global interpretations.

The latter may reconcile the seeming discrepancy between the findings from studies focusing on automatic and reflective cognitive processes, suggesting that absence of positive self-bias when learning social evaluation leads to overly negative post-event interpretations (Button et al., 2015).

While these findings were consistent with cognitive models of social anxiety, the socially anxious individuals in our sample also had higher levels of state anxiety. Therefore, we could not exclude the possibility that the social anxiety ‘phenotype’ resulted from differences in state anxiety, rather than arising from differences in the trait-like fears and beliefs characterising social anxiety. Disentangling the influence of state and trait anxiety on social evaluative inference may have important clinical implications informing whether the patient’s treatment should target fluctuations in their state anxiety or target social anxiety beliefs.

Inhaling 7.5% carbon dioxide (CO_2_) is shown to robustly induce a high state of anxiety (Attwood et al., 2014; Garner et al., 2011, 2012), and is often used as a model of Generalised Anxiety Disorder (GAD) (Bailey et al., 2005b, 2007). Breathing 7.5% CO_2_ increases heart rate and blood pressure, as well as subjective reports of anxiety, nervousness, worry, apprehension and fear (Bailey et al., 2005a, 2007). In humans, the 7.5% CO_2_ model increases sensitivity to threatening stimuli (Garner et al., 2011, 2012) and increases negative interpretations of ambiguous social information presented via real-world close circuit television recordings (Cooper et al., 2013). Work in mice suggests a role for the amygdala, as inhaled CO_2_ reduces brain pH and evokes fear responses, and these effects seem dependent on acid-sensing ion channels in their amygdala (Ziemann et al., 2009). However, individuals with bilateral amygdala lesions can still show a strong response to 35% CO_2_ (Feinstein et al., 2013), and rather than potentiating defensive eye-blink startle responses (which are fear responses under the control of the amygdala), a 7.5% CO_2_ challenge reduces their speed and magnitude (Pappens et al., 2012; Pinkney et al., 2014). This latter finding seems at odds with the threat sensitivity and anxiety-inducing effects of CO_2_ inhalation; however, while disorders characterized by focal fear (e.g. specific phobia) show robust fear potentiation, disorders of long-enduring, pervasive apprehension and avoidance, such as depression and GAD (and thus, arguably, social anxiety) show diminished startle responses (McTeague and Lang, 2012). Furthermore, the startle response potentiation during an interoceptive threat, such as mechanically-resisted breathing, seems dependent on attentional direction (Pappens et al., 2011), implicating a modulatory role of cognitive load (Pinkney et al., 2014). Consistent with this, CO_2_-induced state anxiety is associated with increased errors in identifying faces (Attwood et al., 2013) and may impair speech perception, potentially via distraction (Mattys et al., 2013).

Turning to other models for increasing state anxiety, it was found that the social stress challenge facilitates processing of negative, but not positive, emotional information and it consistently impairs working memory (Luethi et al., 2008; Schoofs et al., 2008). To our knowledge, no previous studies have investigated the effects of inducing anxiety, via 7.5% CO_2_ inhalation, on learning social evaluation.

The aim of this study was to investigate the role of state anxiety on social evaluation learning by using the 7.5% CO_2_ inhalation method to experimentally induce state anxiety in conjunction with the social evaluation learning task (Button et al., 2015), in a sample of healthy volunteers. The task is a probabilistic learning task that assesses the ability to learn four social evaluation rules: self-like, self-dislike, other-like and other-dislike. In line with previous research (Button et al., 2015), we expected to find a strong preference for learning the positive, relative to negative, evaluation and that this positive bias would be strongest for the self (i.e. positive self-bias).

We hypothesised that increasing state anxiety would increase sensitivity to social-threat, manifested as decreasing the study subject’s positive (and increasing the negative) responses across all rules in the social evaluation learning task. If supported, this would indicate that simply increasing state anxiety via the 7.5% CO_2_ model would not be sufficient to account for the effects of social anxiety on evaluative learning; however, we thought that increasing state anxiety in those with social anxiety traits might exacerbate the effects of social anxiety, as the threat attributed to self-negative evaluation would increase. Thus, a second hypothesis was that increasing state anxiety would exaggerate any association between social anxiety and self-referential learning; however, as the study sample was unselected for trait anxiety, we were likely to be underpowered to test trait effects. To reduce model complexity and in an aim to boost power, we therefore restricted this analysis to a single rule *a priori*, self-dislike, where the association with social anxiety was previously found to be the strongest (Button et al., 2015).

## Methods

The full methods used in this study are provided in the protocol registered on the Open Science Framework (https://osf.io/yig9n/).

### Participants

Healthy volunteers were recruited via email from an existing mailing list of individuals who have consented to be contacted about research studies, by word of mouth, at the university job shop or by advertisement on and around the university precinct. Potential participants completed an online screening questionnaire that assessed them for study inclusion and exclusion criteria. Inclusion criteria comprised being between 18 and 50 years of age, with English as a first language or an equivalent level of fluency.

Exclusion criteria comprised:

- Alcohol consumption < 36 hours prior to the study;
- Not currently being registered with a general practitioner;
- Current use of illicit drugs;
- Systolic or diastolic blood pressure > 140/90 mmHg;
- Heart rate at < 50 or > 90 beats per minute (bpm);
- Female gender subjects who were pregnant or breastfeeding;
- Those with a body mass index (BMI) < 17 or > 30 kg/m^2^;
- Having a significant current or past medical or psychiatric illness;
- A strong personal or family history of a mood disorder, including panic disorder;
- Having an ongoing physical illness or abnormality (e.g. a history of cardiac or respiratory problems, including asthma);
- Having a personal history of migraine headaches requiring treatment;
- Drinking > 35 units/week if of female gender or 50 units/week if of male gender (where one unit equals one 25-mL single measure of a spirit at 40% alcohol by volume, or one-third of a pint of beer (5–6% alcohol by volume) or one-half a standard (175 mL) glass of red wine (12% alcohol by volume);
- Being regular (i.e. daily) cigarette smokers;
- Drinking more than eight caffeinated drinks per day;
- A personal history of alcoholism or drug dependence;
- Medication use (except as a local treatment, aspirin or paracetamol) within the past 8 weeks;
- Having impaired or uncorrected vision;
- Having hearing problems and/or hearing aids.

This study was approved by the Faculty of Science Research Ethics Committee at the University of Bristol, UK (# 2905148227). We conducted our study according to the revised Declaration of Helsinki (2013) and good clinical practice guidelines; participants gave consent to have their anonymised data made publically available (DOI: 10.5523/bris.1wc6gtbaujq5p1i56fz2vqdmq4).

### Materials

As described elsewhere (Button et al., 2012, 2015), the chosen social evaluation learning task used 64 word pairs comprised of positive and negative words, selected from personality trait descriptors (Anderson, 1968).

The Brief Fear of Negative Evaluation Scale (BFNE) is a 12-item, self-rated scale that assesses cognitive aspects of social anxiety (Leary, 1983). The Social Interaction Anxiety Scale (SIAS) and Social Phobia Scale (SPS) are companion measures of social anxiety (Mattick and Clarke, 1998). We assessed trait and state anxiety using the Spielberger State-Trait Anxiety Inventory State (STAI-S) and Trait (STAI-T) sub-scales (Spielberger, 1983).

### Procedures

Participants completed one session lasting approximately 2.5 hours. After providing informed consent, participants were screened to ensure that no significant change (e.g. diagnosis of illness or use of medication) had occurred since the online screening. Participants also provided a urine screen for drugs of abuse (all participants) and pregnancy (female participants), expired breath tests for recent alcohol use and smoking, and readings for blood pressure and heart rate. Their height and weight were also measured, and the participants’ psychiatric health was assessed using a neuropsychiatric interview developed from the Mini-International Neuropsychiatric Interview (MINI) (Sheehan et al., 1998). The purpose was to determine good psychiatric health, and we terminated the study session if there was any indication of symptoms that may be indicative of a psychiatric disorder. Therefore, we administered a truncated version of the MINI, which comprised the primary questions, and which omitted the follow-up questions that aimed to diagnose. Study participants also provided contact details for their current General Practitioner.

Prior to the first gas inhalation, baseline measures of blood pressure (systolic blood pressure/diastolic blood pressure (SBP/DBP)), heart rate and state anxiety were taken, as well as the trait anxiety measures mentioned in the Materials section. Participants were then fitted with an oral-nasal face mask; and they were reminded that the gas might make them feel anxious and that they could stop the inhalation at any time. During each inhalation, participants breathed the gas for 2 minutes prior to beginning the social evaluation learning task. These inhalations lasted for the duration of the task (approximately 10 minutes). Immediately after each inhalation session, we measured the participant’s blood pressure (SBP/DBP) and heart rate, and the participants completed the state anxiety measure. After a 30-minute ‘wash-out’ period, the second inhalation followed an identical procedure to the first, except for the gas used. The order of the gas given (medical air or 7.5% CO_2_) was counterbalanced across the participants. Upon completion of the study, participants were debriefed and reimbursed £20.

The social evaluation learning task is based on probabilistic stimulus-reward learning tasks (Button et al., 2012; Chamberlain et al., 2006), and adapted to incorporate pseudo-social context (Button et al., 2015). The task used in this study is adapted from the task used in Button et al. (2015). The original task comprised six blocks equating to learning six social evaluation rules: self-like, self-neutral, self-dislike, other-like, other-neutral and other-dislike.

As the task was repeated in the two gas inhalations, we decided to shorten the task to reduce participant testing time, by removing the two neutral blocks. Therefore, the task used in this experiment had four rule blocks: self-like, self-dislike, other-like and other-dislike. Excluding the neutral rules also conferred analytical advantages, allowing us to examine learning outcomes, such as errors to criterion (e.g. eight consecutive rule-contingent answers (Button et al, 2012)), which we were unable to investigate in Button et al. (2015), as positive and negative responses are equally correct or incorrect during the neutral evaluation rules. Each block lasted 32 trials. The order of presentation of self/other and, within that, the order of presentation of the like/dislike rule was randomly determined by the random option in e-prime. Before starting, the investigator instructed the participant that they would be meeting a series of four personas, during the four test blocks. Each persona required the participant to learn one of two social rules (person is liked by the persona, person is disliked by the persona) while in one of two conditions, self-referential or other-referential.

In each block, the participant was presented with 32 positive/negative word pairs and they were instructed to select the word in each pair that corresponded most with what the persona thinks about themself, or about the other. In response to feedback as to whether their choice was correct, the participants were to use trial and error to learn whether the persona liked them or the other.

The feedback contingency corresponded to the different rules:

- Like (positive word correct 80%, negative word correct 20% of the time); and
- Dislike (negative word correct 80%, positive word correct 20% of the time).

**Figure 1.**
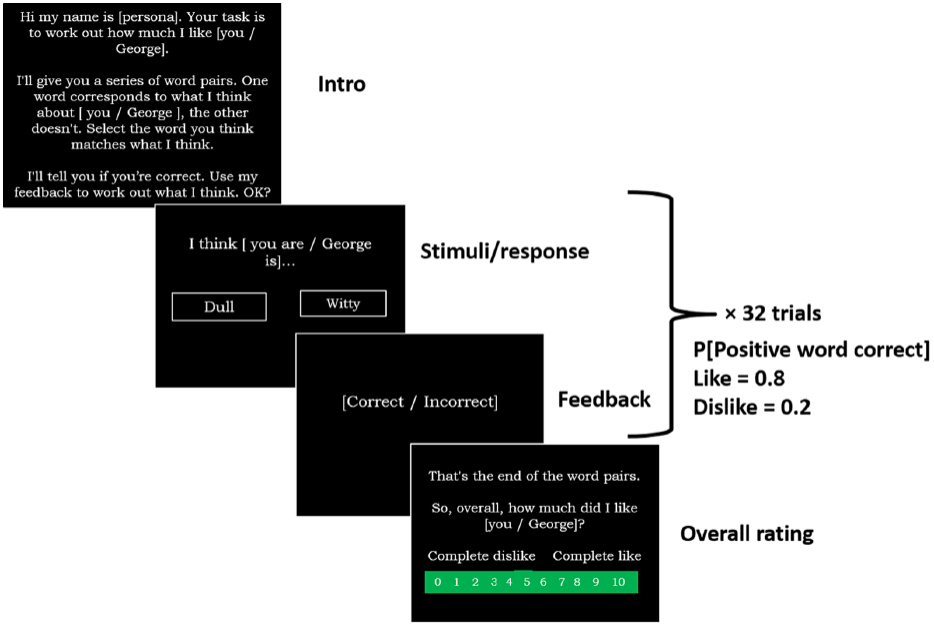
Social evaluation learning task. Block structure repeated four times. For the two self-referential blocks (self-like and self-dislike) ‘you’ is included and ‘George’ omitted from the slides, and vice versa for the two other-referential blocks (other-liked and other-disliked).

Each block ended with the participant rating how much they thought the persona liked either them (self-referential) or the other person (other-referential). Thus, this global rating phase required the participants to reflect on their learning during the previous 32 trials. The block structure of the social evaluation learning task is shown in Figure 1.

### Sample size determination

With a sample size of *n* = 48, we had 80% power at an alpha level of 5% to detect a mean difference corresponding to 6.5 (SD 14) fewer positive responses under the medical air versus 7.5% CO_2_. Therefore, this study was powered to test for effects between 7.5% CO_2_ and air within participants.

### Data analysis

We compared the STAI-S, heart rate and blood pressure scores taken after 7.5% CO_2_ inhalation and air, using paired-sample *t*-tests to check that the CO_2_ manipulation had the expected effect of inducing state anxiety.

The *a priori* study hypothesis that the positive response rate would be lower under 7.5% CO_2_ relative to medical air was examined using random effects Poisson regression (using the command xtmepoisson) in the statistical software package Stata 11 (StataCorp, 2009). The primary outcome measure was the count of positive responses, and the Poisson regression was appropriate for our count data (Vittinghoff, 2005). To give the incidence rate ratios (IRRs), we exponentiated the output, as these are more intuitive to interpret as a percent change (e.g. an IRR of 1.33 corresponds to a 33% increase in positive responses and an IRR of 0.98, a 2% decrease). Therefore, the IRR coefficients indicated the percent change in the positive response rate for each unit increase in the explanatory variable (i.e. gas, condition and rule). We modelled outcome (the count of positive words) as a function of gas (medical air or 7.5% CO_2_), condition (self and other) and rule (like or dislike), to test for the hypothesised main effect of gas on the positive response rate. We then introduced the gas × condition, gas × rule and gas × condition × rule interaction terms into the model, to test for interaction effects.

A positive response rate, while a suitable outcome for assessing bias in valence (e.g. a general negativity / positivity bias), is arguably a poor proxy for assessing learning. We previously used errors to criterion as our learning outcome, calculated as the number of errors made before reaching the criterion of eight consecutive rule-contingent answers (where the criterion is not met, total errors are used), which may be a more sensitive learning measure. In line with Button et al (2012), we therefore repeated the above analyses using errors to criterion as the outcome measure.

We analysed the global ratings using random effects linear regression (using the Stata command, xtmixed), as global ratings are normally distributed. Therefore, the regression coefficients represent the change in rating score for each unit change in the explanatory variable. We modelled outcome (rating) as a function of gas (medical air or 7.5% CO_2_), condition (self or other) and rule (like or dislike), to test for the hypothesised main effect of gas on the positive response rate. We then introduced the gas × condition, gas × rule and gas × condition × rule interaction terms into the model, to test for any interaction effects.

Unlike our previous studies, which selected for extreme BFNE scores (Button et al., 2012, 2015), our present sample was unselected; therefore, BFNE scores clusterd tightly around the population mean, thus reducing our power to test for trait social anxiety effects. Furthermore, tests for interactions are often underpowered, and to test whether the within participant effects of gas differ by trait social anxiety levels would require a substantially larger sample, not least because this would require testing a 4-way FNE × gas × rule × condition interaction. We previously found that social anxiety was associated with fewer positive responses particularly in the self-dislike rule (Button et al., 2015). To maximise power to test our second *a priori* hypothesis, we restricted our analysis to the self-dislike rule, where the social anxiety effects were expected to be greatest. This reduced model complexity; and using random effects Poisson regression, we modelled the positive responses in the self-dislike rule as a function of gas, FNE, and gas × FNE.

## Results

### Characteristics of participants

**Figure 2.**
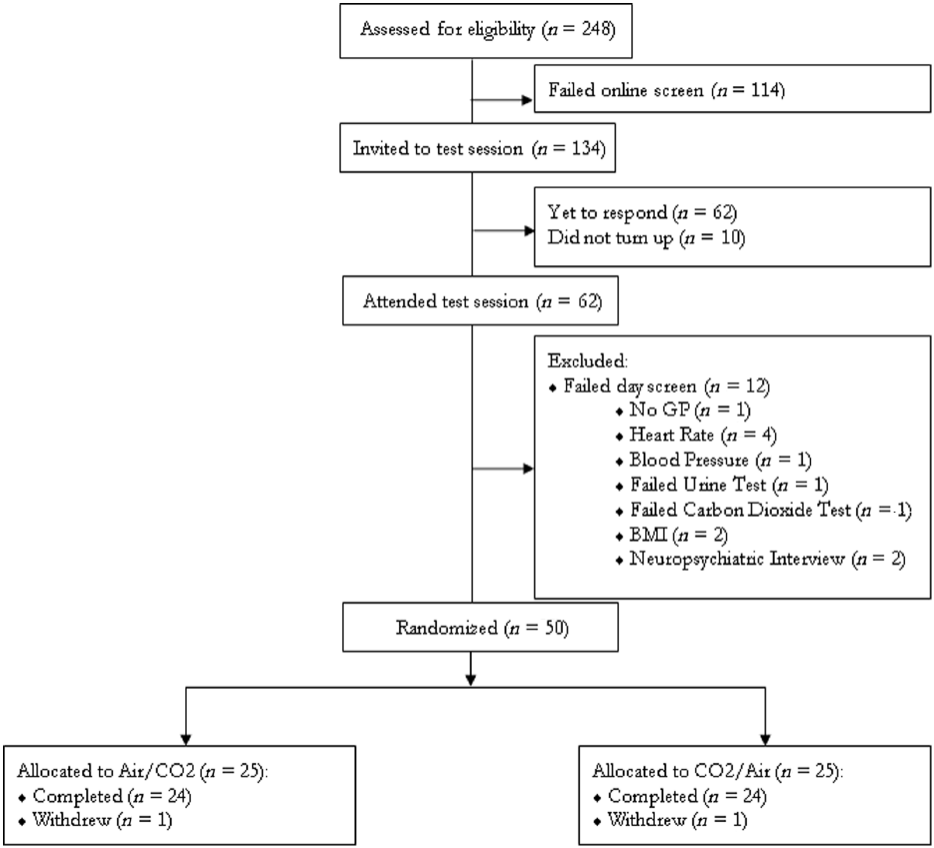
Flow chart of participants through the study. BMI: Body mass index; GP: general practitioner.

We had 48 participants (female participants *n* = 24) complete the testing, providing data for analysis. Their mean age was 23 years old (SD 5; range 18 – 50) and their mean BFNE score was 34.8 (SD 7.0; range 22 – 51). Participant recruitment is shown in Figure 2.

### CO_2_ manipulation check

Relative to air, CO_2_ inhalation increased the participants’ state anxiety (mean difference 20.2; 95% CI 17.4, 29.9; *p* < 0.001), heart rate (mean difference 6.9; 95% CI 4.0, 9.9; *p* < 0.001), and SBP (mean difference 4.0; 95% CI 1.6, 6.5; *p* < 0.001), and had little effect on DBP (mean difference 0.4; 95% CI −1.5, 2.3; *p* = 0.66).

### Descriptive data

Table 1 shows the mean (SD) positive response rates and mean errors to criterion for each rule in CO_2_ and air. Inducing state anxiety via CO_2_ had little effect on self-referential learning, both in terms of positive response rate and errors to criterion (Table 1). By contrast, CO_2_ led to a substantial increase in errors to criterion in the other-referential condition; and thus, fewer positive responses for other-like and more positive responses for other-dislike (Table 1).

**Table 1.** Mean (SD) for the positive response rate (number of positive responses in 32 trials) and errors to criterion by condition-rule and gas.

	Condition-rule	Air (*n* = 48)		CO_2_ (*n* = 48)		Difference
		M	SD	M	SD	
Positive response rate	self-like	0.87	0.34	0.84	0.37	0.03
	self-dislike	0.23	0.42	0.24	0.42	0.00
	difference	0.64		0.60		
	other-like	0.86	0.35	0.78	0.41	0.07
	other-dislike	0.18	0.39	0.26	0.44	− 0.08
	Difference	0.68		0.52		
Errors to criterion	self-like	3.04	3.97	3.13	4.95	− 0.08
	self-dislike	5.38	4.43	5.63	5.91	− 0.25
	difference	2.33		−1.83		
	other-like	3.06	3.82	5.40	6.03	− 2.33
	other-dislike	4.08	4.79	6.56	6.22	− 2.48
	difference	1.02		−1.17		

M: mean.

**Figure 3.**
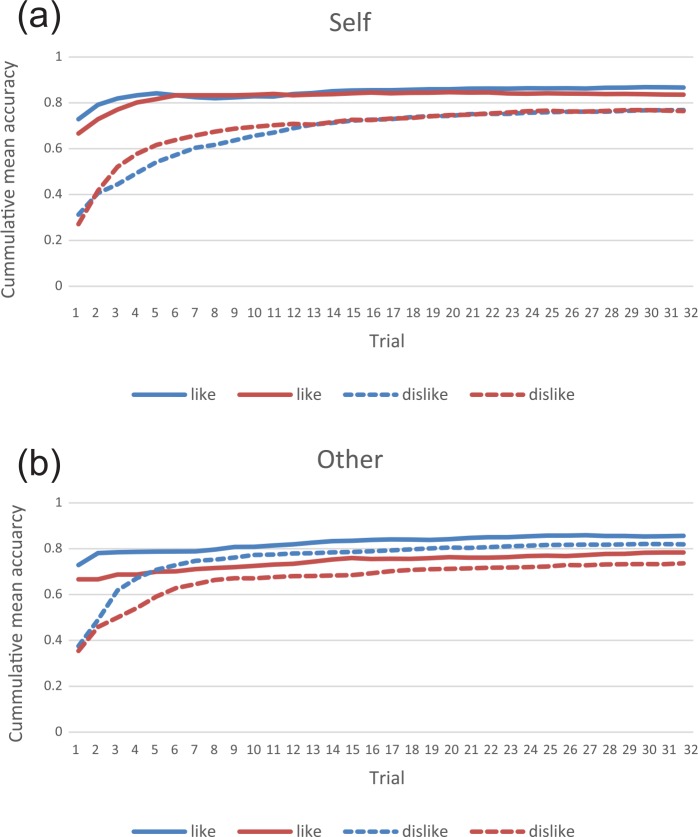
Learning curves for each rule and gas, by condition. (a) Self-condition and (b) Other-condition.

Figure 3 shows the cumulative mean accuracy for the 32 trials for each rule and condition by CO_2_ and air. On average, individuals showed a preference for learning like relative to dislike, which was strongest in the self-referential condition. With regards to gas, the self-referential learning curves were similar for both air and CO_2_. By contrast, other-referential learning seemed more affected by CO_2_-induced state anxiety, with accuracy lower for both like and dislike rules.

### Regression model

Poisson regression modelling the main effects of gas, rule and condition, provided little support for our hypothesis that the positive response rate would be lower under CO_2_, relative to air (IRR 0.99; 95% CI 0.95, 1.04; *p* = 0.8). There was a strong main effect of rule, as expected, with 73% fewer positive responses when learning dislike, relative to like (IRR 0.27; 95% CI 0.26, 0.29; *p* < 0.001), and although individuals on average made 4% fewer positive responses in the self-referential condition relative to the other-referential condition, the CIs included the null (IRR 0.96; 95% CI 0.92, 1.01; *p* = 0.105). Adding the interaction terms into the model found evidence for a gas × rule, and gas × condition × rule interaction (Table 3), which is explained by the decreased and increased positive response rate in the like and dislike rules, respectively, particularly in the other-referential condition (Table 1 shows mean differences). In sensitivity analyses, there was no evidence that the gas order was associated with positive responses (IRR 1.03; 95% CI 0.98, 1.08; *p* = 0.265), and adding gas order into the fully saturated interaction model did not alter the results substantially.

**Figure 4.**
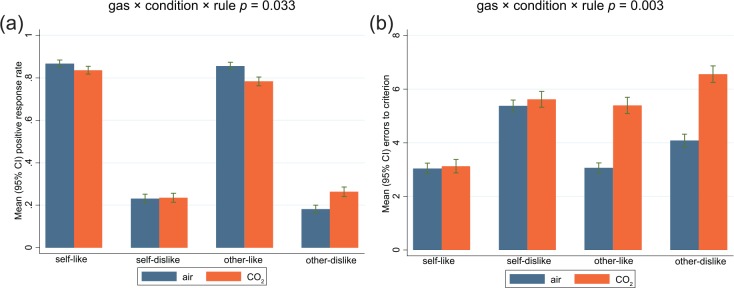
Bar graphs of mean and 95% CI of the (a) positive response rate (left panel) and (b) errors to criterion (right panel) by condition-rule and gas. Positive response rate is lower to other-like and higher to other-dislike in CO_2_ relative to air. This manifests as an increased error rate in the other-referential rules in CO_2_ relative to air.

Examining errors to criterion, we found evidence for the main effects of gas, condition and rule (Table 2). Individuals made 33% (95% CI 21%, 46%) more errors on average during CO_2_ inhalation relative to air, 11% (95% CI 1%, 22%) more errors learning other-referential evaluation relative to self-referential, and 48% (34%, 63%) more errors learning dislike, relative to like. Addition of the interaction terms into the model indicated that the main effect of gas were mostly explained by interactions with gas × condition, and gas × condition × rule interaction (Table 3), suggesting that the increased error rate following CO_2_ is specific to other-referential processing. This is illustrated in Figure 4. In sensitivity analyses, inhaling CO_2_ first was associated with fewer errors than air first (IRR 0.66; 95% CI 0.46, 0.95; *p* = 0.025), but adjusting for order did not substantively alter the results either in the main effects or the interaction model.

**Table 2.** Incidence rate ratios and 95% CIs from regression models testing for main effects of gas, condition and rule. Air, self and like are the reference categories.

	Positive responses				Errors to criterion			
	Coefficient	95% CI		*p*	Coefficient	95% CI		*p*
Gas	0.99	0.95	1.04	0.757	1.33	1.21	1.46	< 0.001
Condition	0.96	0.92	1.01	0.105	1.11	1.01	1.22	0.026
Rule	0.27	0.26	0.29	< 0.001	1.48	1.34	1.63	< 0.001

**Table 3.** Incidence rate ratios and 95% CIs from regression models testing for interactions of gas, condition and rule. Air, self and like are the reference categories.

	Positive responses				Errors to criterion			
	Coefficient	95% CI		*p*	Coefficient	95% CI		*p*
Gas	0.94	0.88	1.02	0.123	0.95	0.77	1.16	0.604
Condition	0.94	0.88	1.01	0.101	0.85	0.74	0.98	0.026
Rule	0.24	0.22	0.26	< 0.001	1.55	1.34	1.79	< 0.001
Gas × condition	0.99	0.89	1.10	0.895	2.03	1.59	2.60	< 0.001
Gas × rule	1.18	1.02	1.36	0.026	1.16	0.91	1.49	0.236
Gas × condition × rule	1.19	1.01	1.40	0.033	0.68	0.52	0.88	0.003

### Modulation by trait social anxiety

We found little support for our hypothesis that state anxiety would exaggerate the association between FNE and positive responses in the self-dislike rule. There was no evidence of a main effect of gas (IRR 1.02; 95% CI 0.88, 1.18; *p* = 0.9), or FNE (IRR 1.00; 95% CI 0.98, 1.02; *p* = 0.99), and no evidence of a gas x FNE interaction, (IRR 1.00; 95% CI 0.98, 1.02; *p* = 0.8). With a sample of 48 people with social anxiety scores that clustered closely around the population mean, we had insufficient power to test further for modulation effects of social anxiety.

### Global ratings

There was no evidence of a main effect of gas, or condition, on global ratings (coefficient − 0.13; 95% CI − 0.38, 0.13; *p* = 0.3; IRR 0.08; 95% CI − 0.17, 0.34; *p* = 0.5; respectively). There was strong evidence for a main effect of rule (coefficient − 4.61; 95% CI − 4.87, – 4.36; *p* < 0.001), with ratings 4.61 points lower for the ‘dislike’ relative to ‘like’ rule, indicating that individuals had learned the rules. In contrast to the online learning phase, there was little evidence that the effect of gas on ratings differed by rule or condition (gas × rule coefficient 0.23; 95% CI − 0.39, 0.85; *p* = 0.5; gas × condition coefficient − 0.17; 95% CI − 0.79, 0.46; *p* = 0.6; gas × condition × rule coefficient 0.58; 95% CI − 0.14, 1.30; *p* = 0.112).

To examine whether responses during the learning phase predicted the global ratings made afterwards, we added positive responses into the main effects model for offline ratings. The number of positive responses predicted offline ratings (coefficient 0.07; 95% CI 0.04, 0.09; *p* < 0.001) with 10 additional positive responses corresponding to a 0.7 increase in global ratings (rating scale dislike 0 – 10 like).

## Discussion

Consistent with the positive self-bias observed in previous research (Button et al., 2012, 2015; De Jong, 2002; Korn et al., 2012), our study participants made more positive responses in the self-condition than the other-condition, and made fewer errors learning self-like than self-dislike; however, contrary to our primary hypothesis, we found that increasing state anxiety did not induce a uniform decrease in positive responses. Instead, positive responses decreased in other-like, increased in other-dislike and remained unchanged in both self-referential rules. In terms of errors, this manifested as an increase in errors following CO_2_ inhalation, in the other-referential rules. Contrary to our second hypothesis, CO_2_ did not influence the association between social anxiety and self-referential learning. Indeed, in this unselected sample, there was no evidence of an association between trait BFNE and positive responses in the self-dislike rule.

### Limitations

As the sample was unselected for trait anxiety, we assumed we would be underpowered to test trait effects. Therefore, we restricted the trait analysis *a priori* to the self-dislike rule, where social anxiety effects were previously found to be the strongest (Button et al., 2015); however, the sample that we recruited clustered more tightly around the population mean BFNE score than expected, thus reducing our power to test for trait effects even further. Although our results suggested that self-referential processing is robust to fluctuations in state anxiety in a ‘low’ anxious population, we cannot be sure this stability in self-referential processing would hold in a highly socially anxious group. Further work is required to examine this. Furthermore, 7.5% CO_2_ inhalation models physiological state anxiety, enabling us to test the effects of increasing physiological arousal and subjective measures of state anxiety on social-evaluative learning. Further work could investigate whether increasing state anxiety via a socially stressful paradigm, such as the Tier Social Stress Test (Kirschbaum et al., 1993) has the same effects on social evaluative learning.

Inducing state anxiety is associated with increased threat processing (Garner et al., 2011) and increased efficiency in the alerting and orienting attention network function (Garner et al., 2012). When inhaling CO_2_, individuals are more inclined to make a threatening interpretation of ambiguous social scenes, when viewing close circuit television (Cooper et al., 2013), and to show a decreased accuracy in identifying facial expressions (Attwood et al., 2013). In light of this, and its use as a model for GAD, we hypothesised that increasing state anxiety via 7.5% CO_2_ inhalation would induce a general negativity bias when learning social evaluation; individuals would be less inclined to choose positive words and more inclined to select negative words. However, we found no evidence to support our hypothesis. Instead, we found that increased state anxiety was associated with increased errors in learning other-referential evaluation. Inducing state anxiety had little influence on self-referential processing. These findings are more consistent with state anxiety impairing working memory (Luethi et al., 2008; Schoofs et al., 2008) and thus reducing accuracy, similar to Attwood et al (2013) for other-referential learning; however, self-referential processing seems robust to such impairments.

Our results show clear evidence of positive self-bias in this healthy and low-anxiety population. Participants made more positive word responses in the self-referential compared to the other-referential condition, and made fewer errors in learning self-like than dislike. These results are consistent with suggestions that positive self-biases are protective for mental health (Taylor and Brown, 1988,1994a, 1994b), and are maintained via preferentially processing the positive over the negative self-relevant information (Korn et al., 2012).

Our results suggested that self-referential processing is robust to changes in state anxiety; inducing state anxiety was not sufficient to induce the loss of positive self-bias associated with social anxiety. This is at odds with the effects of CO_2_ on increasing the sensitivity to threat, and threatening interpretations of ambiguous closed-circuit television videos (Cooper et al., 2013; Garner et al., 2012); however, they are more consistent with findings suggesting that 7.5% CO_2_-induced anxiety interacts with attentional direction and cognitive load (Mattys et al., 2013; Pappens et al., 2011). While the current study design cannot directly test whether state anxiety modulates social learning in highly socially anxious individuals, our results do suggest that previously observed variations in self-referential bias arise at least in part due to trait, rather than state, social anxiety characteristics. Inducing state anxiety is not sufficient to induce the social anxiety phenotype in those without trait social anxiety fears of negative evaluation. Furthermore, they suggested that the 7.5% CO_2_ model of more generalised anxiety may not be useful for modelling social anxiety.

In contrast, increased state anxiety was associated with increased errors in learning in other-referential evaluation. One interpretation of these findings is that self-referential processing recruits a strongly ingrained core belief structure (i.e. self-schemata), and is thus less susceptible to changes in state anxiety. Self-relevant information is known to receive preferential processing (Humphreys and Sui, 2015) and our findings support the special nature of self-referential processing. By contrast, other-referential processing likely recruits less established cognitive networks and may thus be more susceptible to the influence of state anxiety, potentially via impairing working memory (Luethi et al., 2008; Schoofs et al., 2008). This is particularly likely in the current paradigm, where the ‘other’ is a fictional character called George, of whom participants had little prior knowledge or beliefs to base their learning on.

In this study, positive self-referential bias was only evident in the online learning phase, and not in the offline learning phase. Dual process models functionally distinguish between automatic, online processes and the more reflective processes assessed offline (Strack and Deutsch, 2004). In support of this, we had previously found that self-referential offline ratings tend to be more negative than the online responses. For example, in extremely high socially anxious individuals, an absence of positive self-referential bias online (i.e. similar positive response rate for self- and other-referential rules, and accurate relative to true rule contingency) was associated with a negative bias offline (i.e. rating themselves less favourably than others, and less favourably than the true rule contingency). Furthermore, individuals in the mid-range showed a positive self-referential bias online, but no bias offline, as seen in Figure 3 of Button et al. (2015). The results from the current study, where the sample clusters tightly around the population mean for social anxiety, are consistent with this and may reflect the distinct influence of reflective appraisal in line with dual-process models (Strack and Deutsch, 2004).

In conclusion, positive self-bias when learning social evaluation seems robust to fluctuations in state anxiety. In contrast, state anxiety seems to impair learning of other-referential evaluation. This suggested that the previously observed reductions in positive self-bias in social anxiety were due to social anxiety traits, rather than by an increase in state anxiety and arousal.
